# Supplementary material for: The Portiloop: A deep learning-based open science tool for closed-loop brain stimulation
Source: PLoS One. 2022 Aug 22;17(8):e0270696. doi: 10.1371/journal.pone.0270696 (PMC9394839; doi:10.1371/journal.pone.0270696)
Supplement: S1 File — (PDF) [file pone.0270696.s001.pdf]

Supplementary Information

The Portil∞p: a deep learning-based open science tool for closed-loop brain stimulation

Nicolas Valenchon, Yann Bouteiller, Hugo R. Jourde, Emily B.J. Coffey and Giovanni Beltrame

Appendix A
Model-based explanation of sleep spindles

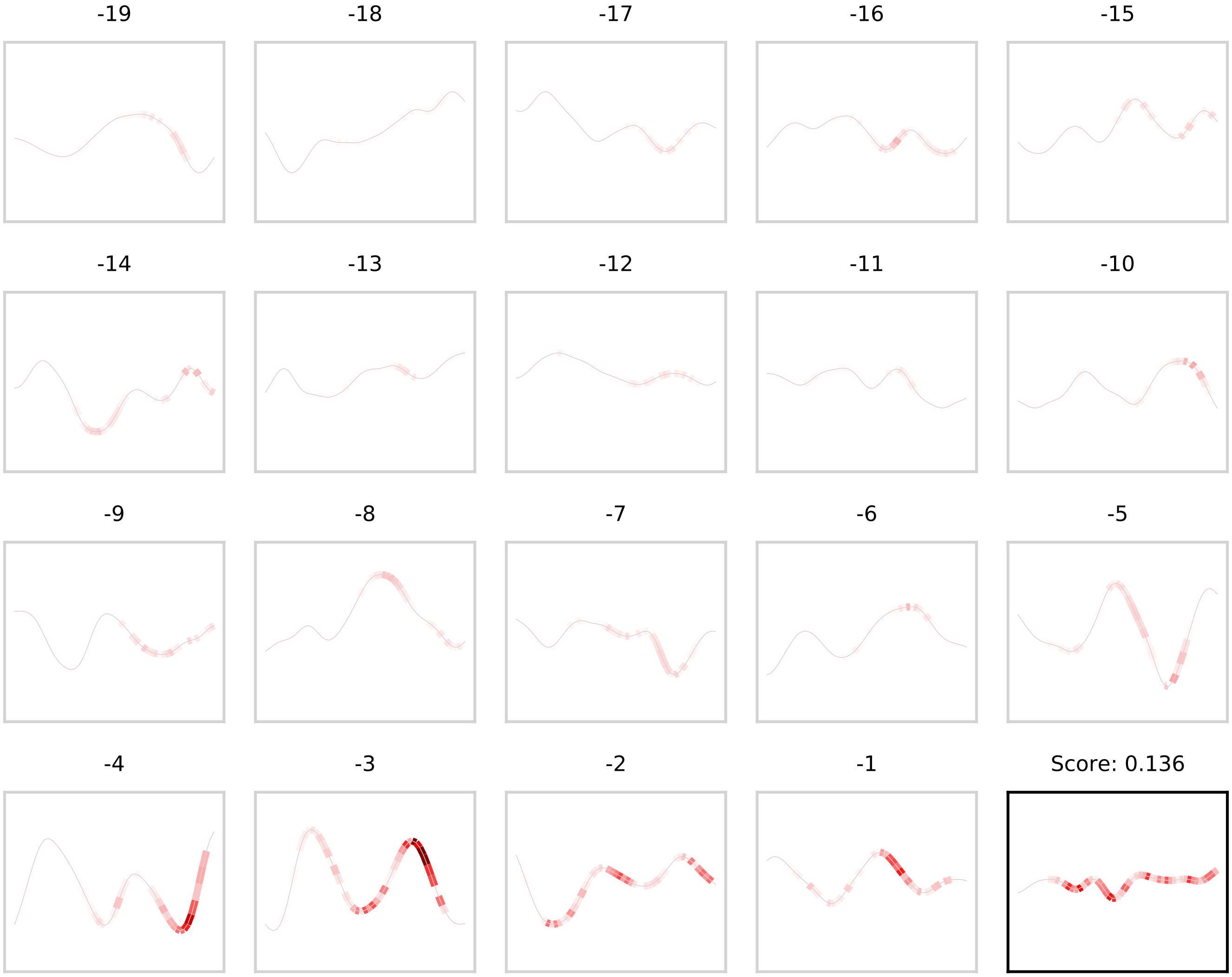

Fig. 1. Integrated gradients (classifier ANN output: 0.136). The *integrated gradients* algorithm enables exploring why the model takes a given decision (the more a portion of the signal is represented in red, the highest its influence on the current output of the ANN). Grey windows are past inputs, whereas the black window is the current input: the past influences the current output due to the RNN. Here, the model finds that it is looking at the aftermath of a sleep spindle. With our time dilation and window size, a small portion of the window overlaps from one sample to the next. We see that this portion (at the left-hand side of each window) is in fact ignored by the model. Therefore, it is probably possible to shrink our model even more, although PMBO did not find this. In the future, this type of visualizations might also help experts better understand what sleep spindles are by revealing unknown influences.

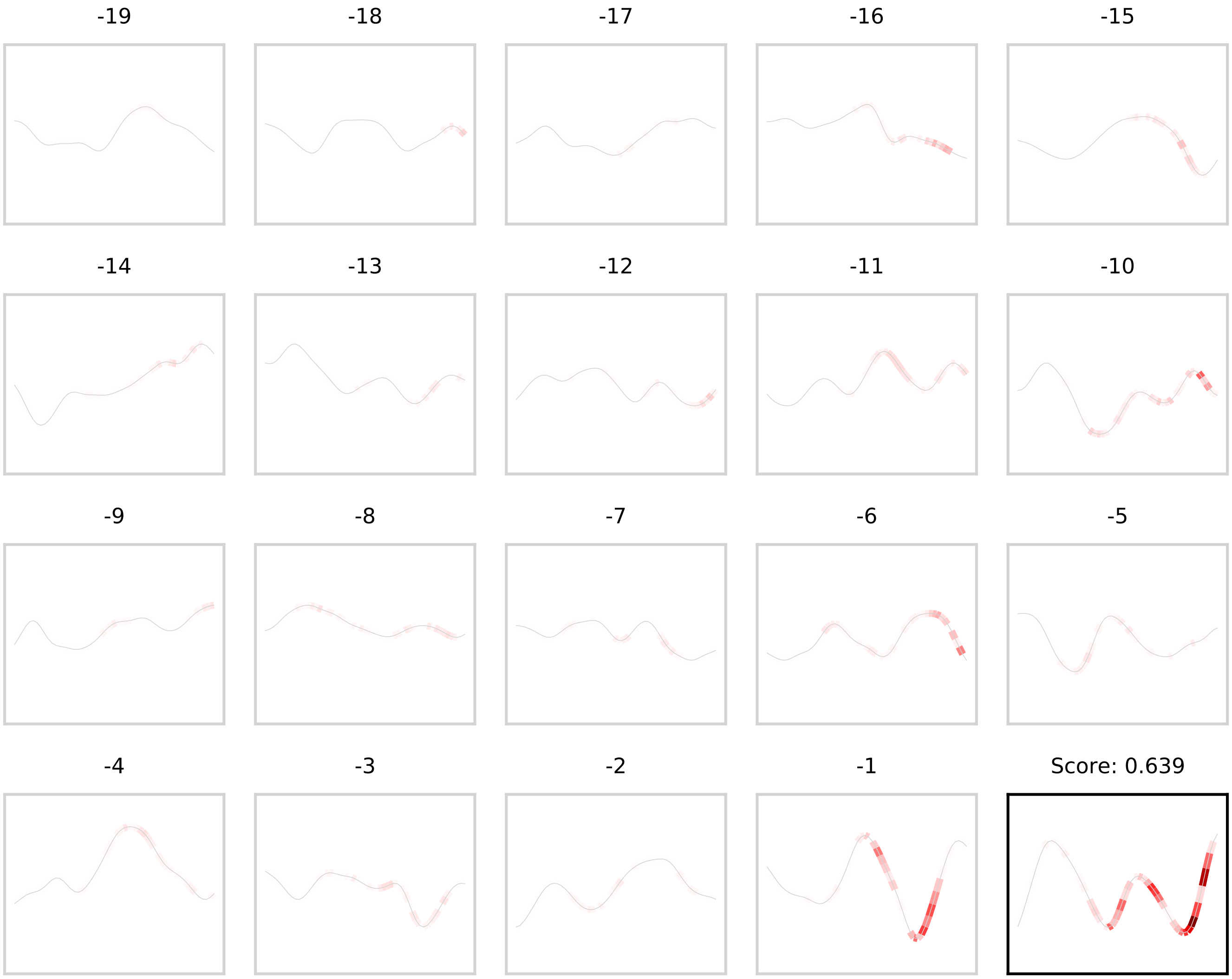

Fig. 2. Integrated gradients (classifier ANN output: 0.639). The current window is within an actual sleep spindle. The model mainly focuses on the spindle itself, but also a few events that happened further back in time, to make its decision.

Appendix B
Stimulation visualizations

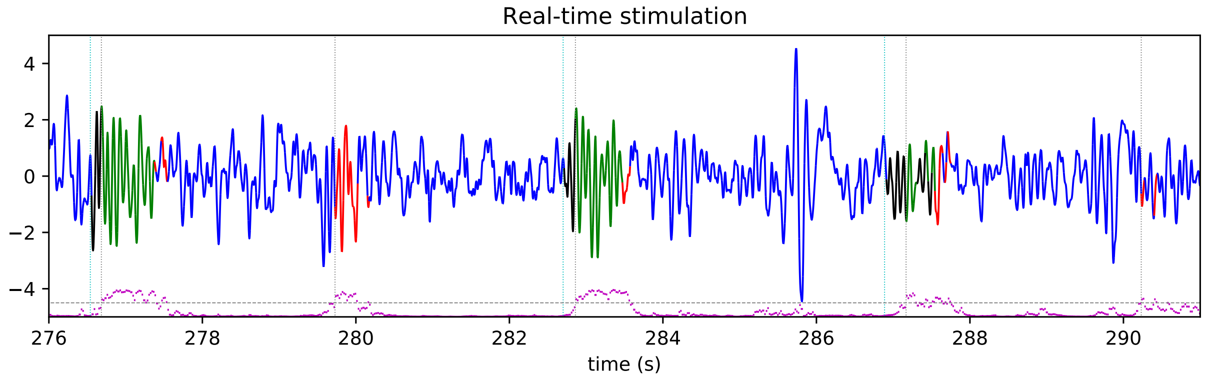

Fig. 3. Classifier with threshold 0.5, overall stimulation performance. Blue: no sleep spindle and no detection. Black: sleep spindle not detected. Green: sleep spindle correctly detected. Red: detection where the signal is not a spindle. Vertical cyan: beginning of a spindle. Vertical grey: stimulus. Magenta: ANN output. Horizontal grey: detection threshold.

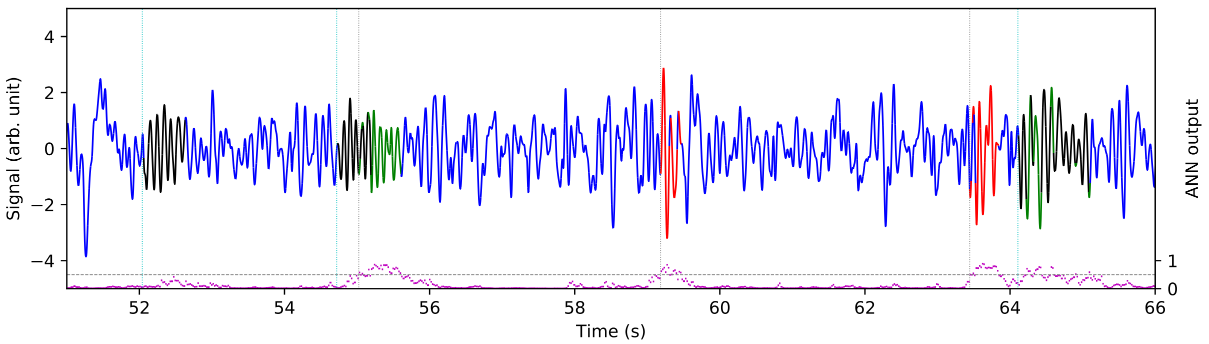

Fig. 4. Different stimulation failure modes (classifier with threshold 0.5). This figure illustrates typical ‘failure’ cases of our final sleep spindle stimulating device. False negative: the first spindle is missed because the threshold is too high. True positive: the second spindle is correctly stimulated. False positive: a part of the signal not labeled as a sleep spindle by MODA is detected as a spindle by the device and stimulated. Almost true positive: the sleep spindle is stimulated in advance (NB: we count this case as a false positive).

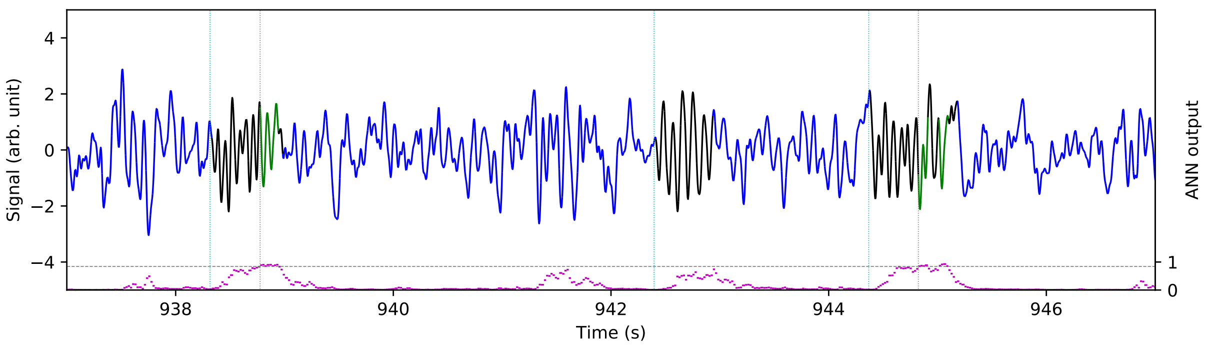

Fig. 5. Classifier with threshold 0.84, failure example. Increasing the detection threshold comes with more false negative stimuli (vertical blue not followed by vertical grey).

Appendix C
PMBO hyperparameters

Table I. Hyperparameters used for PMBO

hyperparameter	selected value
range cost hardware	1000-80000
noise type 1	0,25
noise type 2	0,1
m	200
meta network type	MLP
# layers meta network	3
hidden size meta network	200
optimizer meta network	SGD
learning rate meta network	0.05
weight decay meta network	0.01

Two types of noise are used in our implementation of PMBO to foster exploration of the hyperparameter space:

- noise type 1: portion of the m sampled models that are sampled randomly in the whole hyperparameter space, instead of in a Gaussian around the last completed experiment.
- noise type 2: portion of the time when a model is sampled randomly in the m models, instead of being selected by its Pareto efficiency.

The ANN used for our meta model is a simple Multi-Layer Perceptron (MLP) of 3 fully connected layers. The hyperparameters we use in PMBO are summarized in Table I.

Appendix D
Model hyperparameters

Table II. Hyperparameters used to train the final model

Hyperparameter	Selected value	PMBO
Training		
optimizer	AdamW	
# epochs max	150	
epochs before early stopping	20	
early stopping running average factor	0,1	
batches per epoch	1000	
batch size	256	X
dropout on first layer	False	
dropout factor	0,5	
adam learning rate	0,0005	X
adam weight decay	0,01	
balancing mode	oversampling	
type of training	classification	
sequence length	50	
Architecture		
window size (s)	0,216	X
time dilation (s)	0,168	X
# CNN layers	3	X
# CNN channels	31	X
stride convolution	1	X
kernel size convolution	7	X
dilation convolution	1	X
stride max pooling	1	X
kernel size max pooling	1	X
dilation max pooling	1	X
RNN layers	1	X
RNN hidden size	7	X

The hyperparameters we use in our final model are listed in Table II. Hyperparameters that were chosen by PMBO are marked with a cross under the PMBO column.

Appendix E
Best threshold for classifiers and regressors

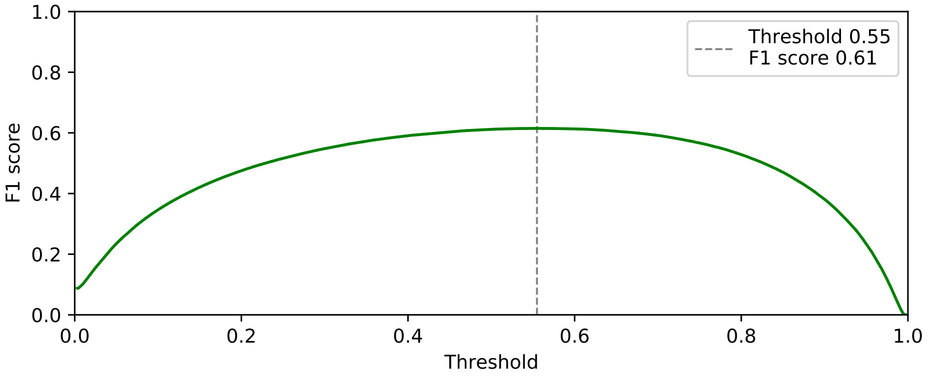

Fig. 6. F1 score evolution with threshold variation on classification

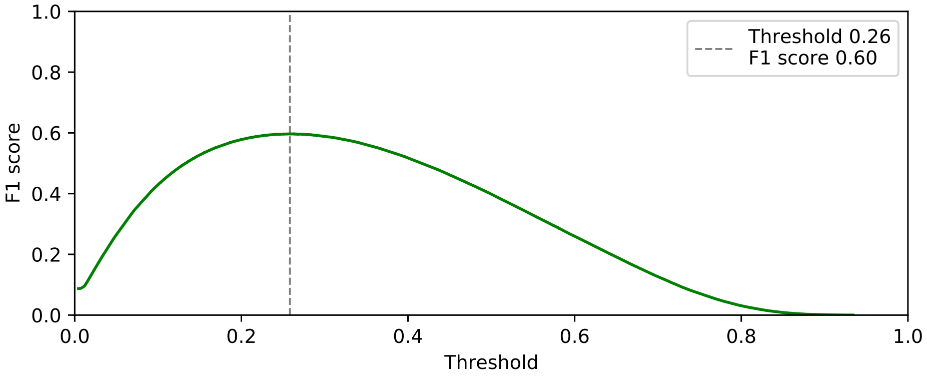

Fig. 7. F1 score evolution with threshold variation on regression

Appendix F
Detection delay distributions

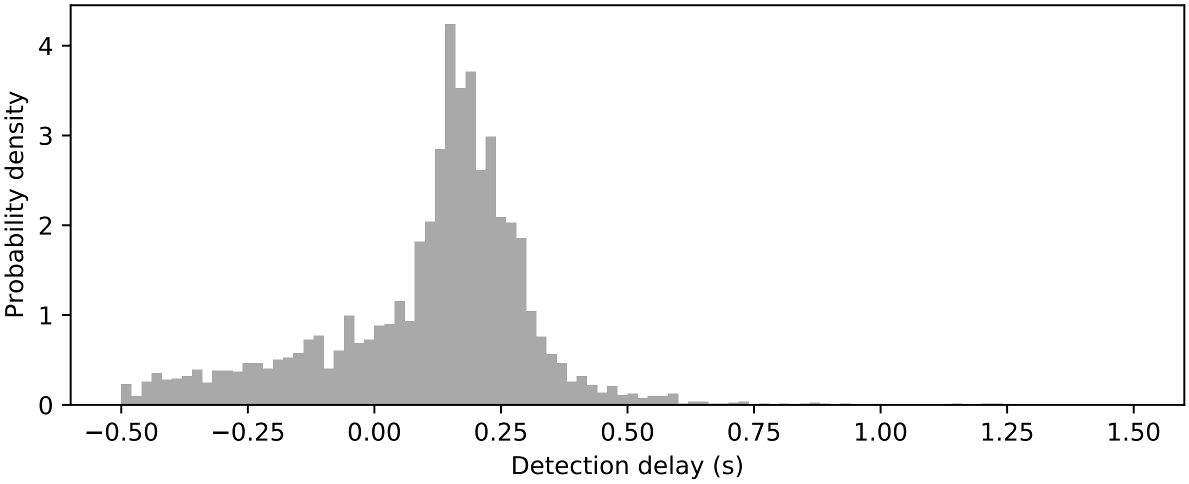

Fig. 8. Stimulation histogram for 1 input network classification with a 0.25 threshold

APPENDIX G
2-INPUT NETWORK ARCHITECTURE

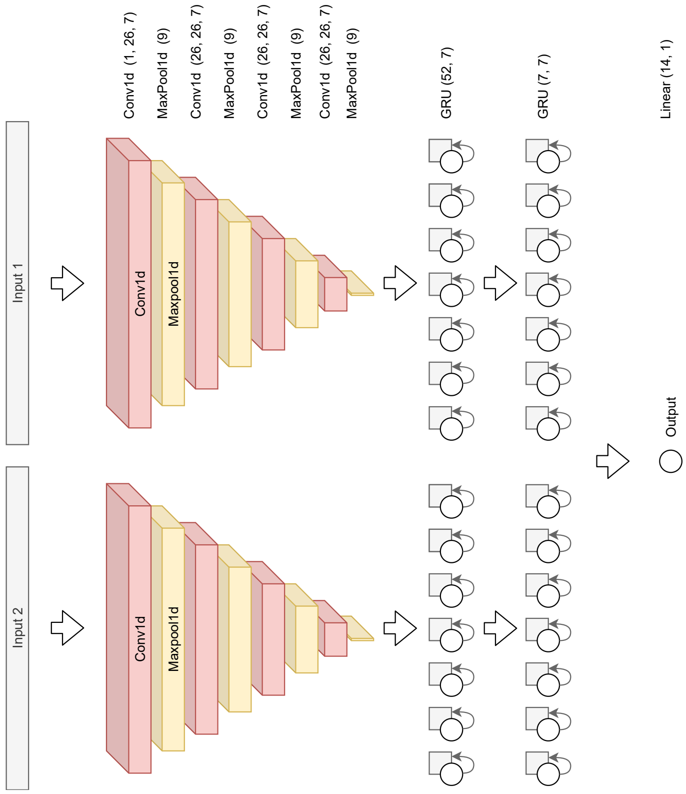

Fig. 9. 2-input neural network architecture. This architecture consists of two sequences of CNN and RNN. The first sequence processes the cleaned raw signal (input 1), whereas the second processes the envelope (input 2). The latent features from both branches are concatenated and fed to a fully connected layer to yield the output of the ANN.

APPENDIX H
THRESHOLD TUNING EFFECT ON STIMULATION

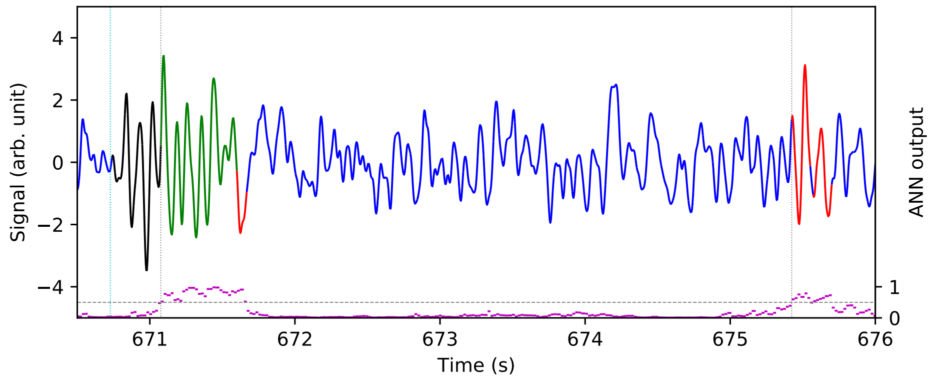

Fig. 10. **Stimulation with a 0.5 threshold.** The first part of the spindle is not detected (false negative), which introduces an ANN software delay. In addition, a small portion of the signal after the spindle is still detected as such (false positive), but it has no impact on our stimulation procedure. Finally, another portion of the signal that was not annotated as a spindle by MODA is detected as a spindle by our model (false positive).

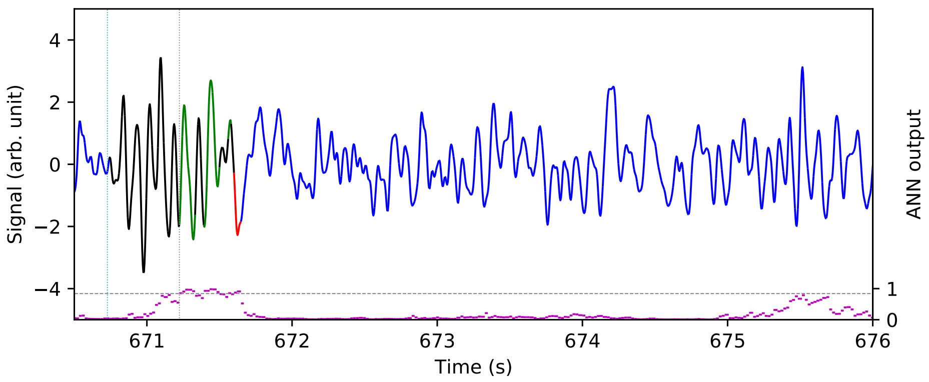

Fig. 11. **Stimulation with a 0.84 threshold.** Increasing the threshold removes false positives at the price of some stimulation delay.

Appendix I
Online standardization through exponential moving average

We standardize the signal on the fly though exponential moving average. In other words, we transform the filtered signal $s(t)$ to $s'(t)$ according to:

$$s'(t) = \frac{s(t) - \hat{\mu}(t)}{\hat{\sigma}(t)} \tag{1}$$

where $\hat{\mu}$ and $\hat{\sigma}$ are estimates of the mean and standard deviation of the filtered signal, computed as follows:

$$\delta(t) = s(t) - \hat{\mu}(t-1) \tag{2}$$

$$\hat{\mu}(t) = \hat{\mu}(t-1) + \alpha_\mu \delta(t) \tag{3}$$

$$\hat{\sigma}^2(t) = (1 - \alpha_\sigma)(\hat{\sigma}^2(t-1) + \alpha_\sigma \delta^2(t)) \tag{4}$$

$$\hat{\sigma}(t) = \sqrt{\hat{\sigma}^2(t)} \tag{5}$$

α_μ and α_σ being hyperparameters in $[0,1]$. This custom real-time standardization makes the signal comparable from one subject to another, enabling generalizable learning.

Appendix J
Parallel Model-Based Optimization

When developing a novel Portiloop application, the practitioner needs to devise a neural network that is both high-performance and lightweight, by selecting the right set of hyperparameters H in the space of all possible hyperparameter sets $\mathcal{H}$. Such hyperparameters include the size of the sliding window, the number of layers in each part of the ANN, the width of each layer, the time dilation (see Section K), the type of optimizer, the hyperparameters of the optimizer itself, etc. $\mathcal{H}$ can be very large, and finding a set of hyperparameters that yields a high-performance model within given hardware constraints is far from trivial. We introduce "Parallel Model-Based Optimization" (PMBO), a network-based algorithm that automates this process in a parallel fashion. Released as open-source along with our code, PMBO is essentially a parallelized and evolved version of "Probabilistic SMBO" [1]. PMBO is a guided-search approach that finds a suitable set of hyperparameters rapidly. For this matter, it uses one machine (or process) to train a *meta network* whose role is to predict non-trivially available costs for any given set of hyperparameters. Furthermore, PMBO uses any available machines (or processes) in parallel to train ANNs from sets of hyperparameters selected based on the cost estimated by the meta model.

The purpose of PMBO is to find Pareto-optimal sets of hyperparameters that minimize both a *software cost* and a *hardware cost*. Hyperparameter selection is a bi-objective problem in our setting: on one hand, we want an ANN that performs well at detecting the desired patterns. We measure this performance in terms of the f1-score of our model. The f1-score depends both on the precision (how sure we are that positive outputs are true positives) and on the recall (how sure we are that we capture all true positives) of the model. It is defined as:

$$\text{f1} = \frac{2 * \text{recall} * \text{precision}}{\text{recall} + \text{precision}} \tag{6}$$

where

$$\text{precision} = \frac{\text{true positives}}{\text{true positives} + \text{false positives}} \tag{7}$$

and

$$\text{recall} = \frac{\text{true positives}}{\text{true positives} + \text{false negatives}} \tag{8}$$

The f1-score is in $[0, 1]$, with 1 being a perfect classifier. We cast this into a minimization problem by defining our *software cost* as $L_s = 1 - \text{f1}$.

On the other hand, we want our ANN to be as lightweight as possible, so it fits in the limited memory of the Portiloop and executes as quickly as possible. A precise measurement of the execution duration and amount of memory needed on the board is difficult. In fact, we do not have access to these results until the model is actually synthesized on the board, which is a lengthy process. Thus, we use the number of trainable weights in the ANN as a proxy for these concerns, and call this number our *hardware cost* L_h. Note that our choice of costs is arbitrary and other custom costs can be used instead.

Let us consider a meta dataset $\mathcal{E}$ of previously completed experiments (*i.e.*, tuples $E = (H, L_s, L_h)$ of hyperparameter sets with their real costs). Let us also consider the following Pareto-dominant relation:

Definition 1. *Let $E = (H, L_s, L_h)$ and $E' = (H', L'_s, L'_h)$ be two experiments in $\mathcal{E}$. We say that E Pareto-dominates E' and we denote $E < E'$ when $L_s < L'_s \wedge L_h < L'_h$. (NB: in the context of this minimization problem, dominating means having the smallest costs, hence the notation)*

For a given experiment E and meta dataset $\mathcal{E}$, we denote $D_{\mathcal{E}}(E)$ as the number or experiments in $\mathcal{E}$ that are Pareto-dominated by E, and $d_{\mathcal{E}}(E)$ as the number of experiments in $\mathcal{E}$ that Pareto-dominate E. In other words:

$$D_{\mathcal{E}}(E) = |\{E^i \in \mathcal{E}, E^i > E\}| \tag{9}$$

$$d_{\mathcal{E}}(E) = |\{E^i \in \mathcal{E}, E^i < E\}| \tag{10}$$

For a given set of hyperparameters $H \in \mathcal{H}$, we further define an estimate of the corresponding completed experiment E as $\hat{E} = (H, \hat{L}_s, \hat{L}_h)$, where $\hat{L}_s$ and $\hat{L}_h$ are estimates of the real costs, computed by the meta network. Note that in our setting, $\hat{L}_h = L_h$ is available and thus only $\hat{L}_s$ is estimated by the meta network. We propose the heuristic Pareto efficiency $\eta(\hat{E})$:

$$\eta(\hat{E}) = a_{\mathcal{E}}(\hat{E}) + b_{\mathcal{E}}(\hat{E}) + s_{\mathcal{E}}(\hat{E}) - h_{\mathcal{E}}(\hat{E}) \tag{11}$$

where $a_{\mathcal{E}}(\hat{E})$ promotes hyperparameter sets whose predicted costs are not dominated by many experiments in $\mathcal{E}$:

$$a_{\mathcal{E}}(\hat{E}) = 1 - \frac{d_{\mathcal{E}}(\hat{E})}{|\mathcal{E}|} \tag{12}$$

$b_{\mathcal{E}}(\hat{E})$ promotes hyperparameter sets whose predicted costs dominate many experiments in $\mathcal{E}$:

$$b_{\mathcal{E}}(\hat{E}) = \frac{D_{\mathcal{E}}(\hat{E})}{|\mathcal{E}|} \tag{13}$$

$s_{\mathcal{E}}(\hat{E})$ promotes hyperparameter sets whose predicted software costs are better than the best software cost amongst all completed experiments in $\mathcal{E}$:

$$s_{\mathcal{E}}(\hat{E}) = \frac{\min(\{L_s, (H, L_s, L_h) \in \mathcal{E}\})}{\hat{L}_s} \tag{14}$$

and $h_{\mathcal{E}}(\hat{E})$ penalizes hyperparameter sets that have a high density with respect to experiments present in $\mathcal{E}$ in terms of their hardware cost. More precisely, we define a range of hardware costs we are interested in, and we split this range into a number of bins. We then compute the binned density of experiments in $\mathcal{E}$ over this range, and multiply this density by the range's width. The penalty $h_{\mathcal{E}}(\hat{E})$ is the height of the resulting bin where $\hat{L}_h$ stands. Multiplying the density by the range's width enforces $h_{\mathcal{E}}(\hat{E}) > 1$ in regions of high density and $h_{\mathcal{E}}(\hat{E}) < 1$ in regions of low density.

Fig 12 explains our PMBO algorithm. A central meta learner is communicating with n peripheral workers to find a hyperparameter set $H^* \in \mathcal{H}$ that is Pareto-optimal for both the software and hardware costs (*i.e.*, non-Pareto-dominated by any other set). For this matter, the algorithm uses a meta dataset $\mathcal{E}$ of tuples $E^i = (H^i, L^i_s, L^i_h)$ to train a meta network that maps any hyperparameter set $H \in \mathcal{H}$ to its corresponding (estimated) costs $\hat{L}_s$ and $\hat{L}_h$ [1]. Once the meta network is trained, it is used to guide the sampling process. More exactly, we sample m hyperparameter sets in $\mathcal{H}$ from a multivariate Gaussian distribution around the hyperparameter set corresponding to the last results received from the workers by the meta learner. Sets that don't satisfy user-defined constraints (*e.g.*, that have already been tested, or, when L_h is available, that fall outside the range we are interested in) are discarded and resampled. We then select the best set in terms of Pareto efficiency, estimated thanks to the trained meta network. This selected set is appended to a buffer, waiting to be consumed by an idle worker. The sampling process is repeated until the buffer is full. When a worker is idle, it fetches an ANN architecture and training instructions from the buffer. The worker yields a measurement of the real hardware and software costs for the current hyperparameter set, which are sent back to the meta learner and appended to the meta dataset. The meta learner then uses the updated meta dataset to train a new meta network, and so on.

[1] $\hat{L}_h$ is an output of the meta network in the general case. However, with our choice of hardware cost it is not, since the ground truth L_h is available.

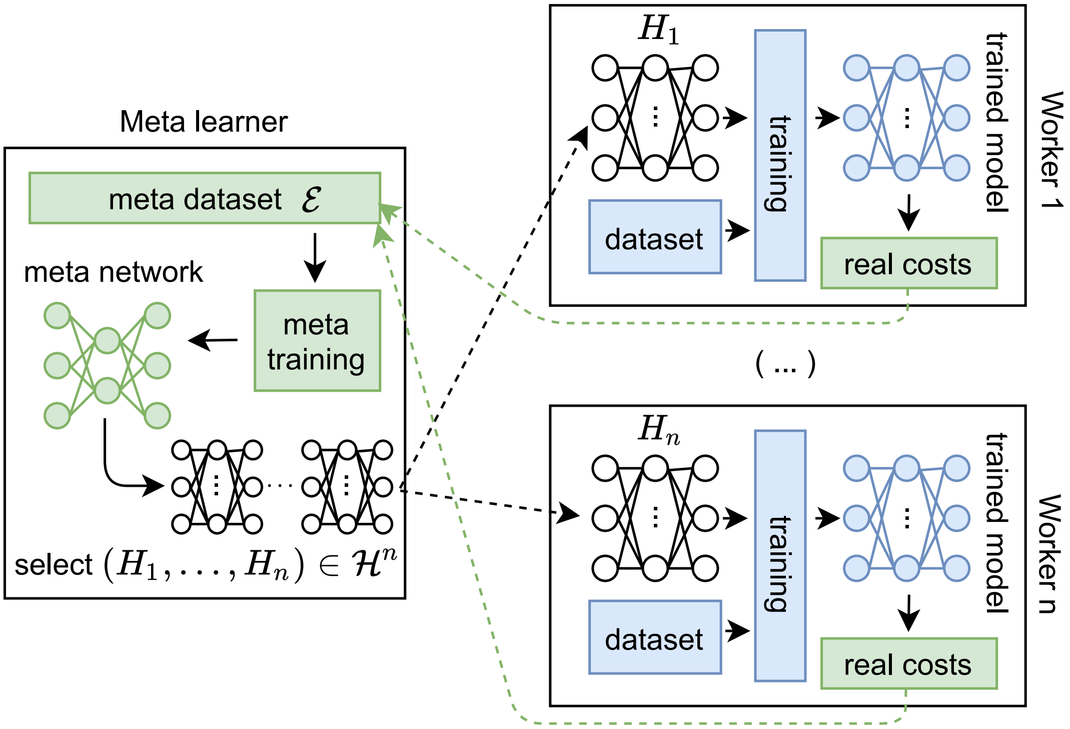

Fig. 12. **The Parallel Model-Based Optimization (PMBO) algorithm.** The algorithm serves to automate the selection of hyperparameters within given hardware constraints. It is based on a standard single-producer and multiple-consumers architecture. A "meta learner" is in charge of producing relevant hyperparameter sets in a guided fashion. It sends these sets to "workers", and keeps producing new sets as long as idle workers remain. Each worker that has received a new set starts training an ANN corresponding to the assigned set. Once this training ends, the real costs of the hyperparameter set can be computed and are sent back to the meta learner. The best-performing models can then be selected for implementation.

APPENDIX K
VIRTUAL ANN PARALLELIZATION WITH TIME DILATION

Given the Portiloop's design constraints, we sought a lightweight means of allowing our resource-restricted network to use as much signal history as possible (as do larger neural networks). Time dilation [2] is a technique that enables recurrent units such as Gated Recurrent Units (GRUs) to look further back in time before gradients vanish, at no computational cost. We propose a version of this technique that allows us to virtually parallelize a single physical ANN into several decoupled virtual models. Our approach enables shallow recurrent neural networks to look further back in time by skipping the redundant information that is inherent to the use of a sliding window as input, while still acting as fast as possible. Fig 13 (a) illustrates how time dilation can be used to look further back in time and avoid redundancy.

Although time dilation enables reaching further back in time at no extra computational cost, this comes with a cost in terms of delays. Since our technique causes samples to be skipped between forward passes in the ANN, a detection delay that can be as long as the time dilation is introduced. We correct this issue by implementing a trick that we call *virtual parallelization*. We create a First In First Out (FIFO) list as large as the time dilation, and fill this list with independent hidden states[2]. At each time-step, we pop a hidden state from this list, feed it to the recurrent units of our ANN, perform a forward pass, and append the resulting hidden state to the list. Doing this without skipping samples is equivalent to having several decoupled models running in parallel as illustrated in Fig 13 (b), although one single ANN is physically used. This trick allows us to keep acting as fast as possible since it removes the need for skipping samples, while still reaching far back in time at no extra computational cost.

[2] *Hidden states* are memory cells of recurrent units like GRUs.

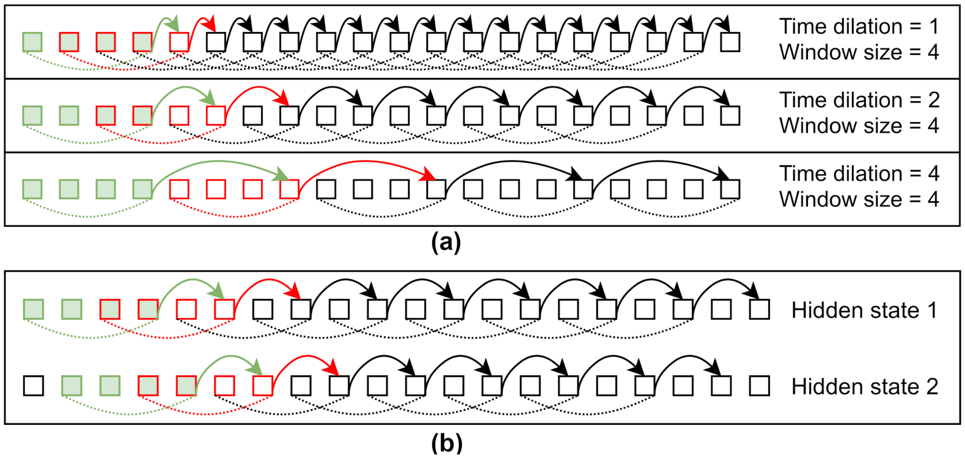

Fig. 13. **A lightweight solution to using historical signal information.** To “look” farther back in time, which facilitates detection accuracy, we introduce (a) Time dilation, and (b) Virtual parallelization. In (a), a sliding window of the 4 last samples (dotted curves) is used as input to the model. The time dilation (arrows) is the number of samples between two forward passes in the ANN. When it is small (top), two consecutive windows overlap (see *e.g.*, green and red windows), meaning consecutive passes contain redundant information. When the time dilation is large (bottom), this issue is corrected, and back-propagation will reach much further back in time for the same number of forward passes. Virtual parallelization reduces delay. In (b), the time dilation is 2, so we keep track of 2 independent hidden states and feed these alternately to the ANN. This trick removes the delay of time dilation.

References

[1] Z. Yin, W. Gross, and B. H. Meyer, “Probabilistic sequential multi-objective optimization of convolutional neural networks,” in *2020 Design, Automation & Test in Europe Conference & Exhibition (DATE)*. IEEE, 2020, pp. 1055–1060.

[2] S. Chang, Y. Zhang, W. Han, M. Yu, X. Guo, W. Tan, X. Cui, M. Witbrock, M. Hasegawa-Johnson, and T. S. Huang, “Dilated recurrent neural networks,” *arXiv preprint arXiv:1710.02224*, 2017.
