# Supplementary material for: The Portiloop: A deep learning-based open science tool for closed-loop brain stimulation
Source: PLoS One. 2022 Aug 22;17(8):e0270696. doi: 10.1371/journal.pone.0270696 (PMC9394839; doi:10.1371/journal.pone.0270696)
Supplement: Attachment — Submitted filename: Response to Reviewers.pdf [file pone.0270696.s002.pdf]

ANSWERS TO THE REVIEWERS' COMMENTS

We would like to thank the editor and the reviewers for their work and comments on the manuscript. We have significantly reworked the manuscript such that the introductory sections should be clear and approachable for readers of both neuroscientific and engineering orientations, and we provide guidance for readers with different backgrounds so that they might direct their attention to the more detailed sections most relevant to their interests. We have also moved some of the technical details to Supplementary Materials and our github repository to reduce length. Please see detailed answers to each reviewer comment, below.

ID	Reviewer's comment	Answer
**Academic Editor's additional comments**		
Thank you for submitting your manuscript to PLOS ONE. After careful consideration, we feel that it has merit but does not fully meet PLOS ONE's publication criteria as it currently stands. Therefore, we invite you to submit a revised version of the manuscript that addresses the points raised during the review process.		
1-1	Please, revise manuscript for clarity, you are requested to do substantial restructuring and shortening of the manuscript.	Thank you for your suggestions. We have significantly restructured the manuscript and have moved some details to Supplementary Information (please see response to reviewers for specific changes). A challenge to our interdisciplinary (neuroscience-engineering) work is that the portion of collaborative projects that is not specific to a reader's discipline can be difficult for them to evaluate and the details are perhaps less interesting to them. However, the two parts are integral to one another: the spindle detection application and its validation is not of interest in fundamental neuroscience separately, but are necessary for neuroscientists to support the device's use and validity in all subsequent works using it. Conversely, the hardware and software designs are not conceptually groundbreaking in isolation, but rather their adaptation and combination in the service of fundamental science is. We believe that both portions are necessary to include and in some level of detail, but have made the text more readable, and have clarified how the work can be useful to audiences with different foci early in the introduction.

(*Continued on next page*)

continued from previous page

ID	Reviewer's comment	Answer
1-2	Please, clearly describe the goal and methods of the study, and provide the details of the design and data analysis.	We have described the goal and approach of the study more clearly in the Introduction and in the Abstract (e.g., from the Abstract: "In this article, we propose the Portiloop, a deep learning-based, portable and low-cost closed-loop stimulation system able to target specific brain oscillations. We first document open-hardware implementations that can be constructed from commercially available components. We also provide a fast, lightweight neural network and an exploration algorithm that automatically optimizes the network model to the desired brain oscillation. Finally, we validate the technology on a challenging test case of real-time sleep spindle detection, with results comparable to off-line expert performance.Software and plans are available to the community as an open science initiative to encourage further development and advance closed-loop neuroscience research")

Reviewer #1

This is a very long and non-stringent, yet potentially very interesting manuscript. I am not able to give an in-depth review at this point, since reading the manuscript is rather confusing, and it would probably take me several days to figure out everything that you are trying to say. I would advise you to do some substantial restructuring and shortening work, then I'll be happy to provide an in-depth review. For the moment, I have some mostly technical remarks:

ID	Reviewer's comment	Answer
1-3	I would not recommend to use a special symbol (infinity) in the title, this will be very difficult for indexing, search engines etc	The infinite sign has been replaced by normal characters.
1-4	Also, it is not the gold standard to share data via a private Google Drive, have you considered using a scientific repository (e.g. Figshare, Harvard Dataverse,...)	Our dataset, models, and all our relevant code are now provided as part of the public Portiloop GitHub repository for convenience (this enables e.g. running our trained neural network out-of-the box and visualizing results in an interactive fashion). The private Google Drive link has been removed.

(*Continued on next page*)

continued from previous page

ID	Reviewer's comment	Answer
1-5	If my computer is counting correctly, the word count is 10,000, which is clearly too long for the manuscript to be read by most people, including myself.<br>It is unclear to me what exactly you are presenting in this paper - you say it's about a new device, but at the same time you talk a lot about machine learning and software benchmarking fundamentals, and sleep physiology, which in my humble opinion is just too much for one manuscript. Please try to be concise and have a clear focus for the manuscript. For example, the best ANN architecture for spindle detection is in principle independent of any hardware implementation, and could therefore be dealt with in a separate paper (or in the Supplementary Material, if you do not deem it worth of a separate paper).<br>Related to this: You open-sourced the software, but how could I get one of these devices if I wanted to? I may have missed it, but I found no information on this. Was it self-built by your lab or did you have an industry partner (which I don't assume since there is on conflict of interest declared). Can I buy it from you (in which case a COI would need to be declared), or am I expected to build it myself, following your instruction (in which case the instruction would have to be much more detailed)? Without at least mentioning any prospective way that I could get hold of one of these devices in the future, I'm not sure how useful a paper describing it will be.<br>by the way, it would be nice to see a picture of the device and how it can be used in the lab environment.	We have considerably restructured and shortened the manuscript, clarified the objectives, and have moved many of the hardware details to Supplementary Information and a Github repository. This is an interdisciplinary work, in the sense that its focus and novelty is on the combination and adaptation of existing technologies to create a research tool for neuroscience. We have provided guidance to readers to help them decide which sections to which to direct their attention, depending on their background and interests. For example, people with the necessary engineering background to build a device will be more interested in the hardware details (note that details are moved to Github), a specialist in neural networks wishing to use or adapt the network for a different neural event might be most interested in the neural network implementation, whereas neuroscientists who are more interested in evaluating the device's research capabilities are likely to get more out of the case study sections (please see the paragraph in the Introduction beginning with "The scope of this work is (...)").<br>We have also clarified what is being offered and who might be able to use it to what ends, throughout (e.g., hardware plans, code for the PMBO algorithm and for the ANN), and we have added pictures of two Portiloop hardware implementations, as suggested.<br>We hope our revised version is more clear about the methodological focus of the manuscript, and that you will enjoy giving it an in-depth review.
1-6	Related to this, I did not find any information about data storage for offline analysis, and how it would integrate with existing lab infrastructures (e.g. Labstreaminglayer).	Thank you for the suggestion; we have added the capability for streaming through LSL and have added in the text, "The Portiloop comes with pre-installed software for recording and visualizing the EEG signal, as well as Python programming interface for the development of extensions or new applications. Practically, the Portiloop can be accessed via WiFi (it acts as a standalone access point and it can connect to an existing network) and it provides a web-based interface based on Python Notebooks that allows to configure the EEG channels, visualize the signals in real-time, start and stop EEG recording (see Figure 3). The recording can be saved either in the internal memory (32GB) or an SD card in EDF format, or streamed through the network using the Lab Streaming Layer (LSL), which guarantees synchronization with microsecond accuracy."

(*Continued on next page*)

continued from previous page

ID	Reviewer's comment	Answer
1-7	This manuscript should not come with 13 figures. Since most of them contain very little information, consider concatenating them to three or four figures with several subplots.	We have combined some of the figures as suggested, and removed some or moved them to Supplementary Information. We have also improved the figure captions so as to more clearly state the purpose of each figure. (We suggest that the final format in which the figures will be embedded in text rather than review format in which they are at the end will make the figures easier to interpret.)
1-8	To sum up, I think closed-loop stimulation is a relevant field with a lack of well-functioning and easy-to-use devices. I would therefore highly appreciate if you could bring your paper into a more digestable shape, so that your work can receive its due appreciation.	Thank your for your support. We hope you will find our revised version much improved, and easy to digest.
**Reviewer #2**		
The Authors developed hardware that can implement Artificial Neural Networks (ANNs) for detecting sleep spindles using FPGAs. Design constraints were imposed to make the hardware lightweight and portable for potential application in close-loop stimulation studies. This study focuses on a deep-learning, open-source toolkit for model-driven design of the FPGA ANNs, Portiloop, using a public sleep dataset (MODA) for training and testing. Such a toolkit could be useful for studies on sleep and brain stimulation, but the results are lacking. I have three concerns.		
2-1	The potential for a toolbox is evident, but such a toolbox's utility and functionality are not demonstrated, but rather implied. The prototypical toolbox distracts from the analyses.	As noted also by Reviewer 1, the scope and goals of the original manuscript were not clear. We have considerably restructured and shortened the manuscript, clarified the objectives, and have moved many of the hardware details to Supplementary Information and a Github repository. The current work focuses on methods development and proof-of-concept using a challenging test case. Our neural network training pipeline, dataset and lightweight pre-trained models are now publicly available on the Github repository of the project. In particular, this enables comparison of our results and using trained models out-of-the box, and we hope will answer your concerns regarding utility and functionality, at least for the software part.<br>The scope is now more clearly summarized in the Abstract (please see also Introduction): "In this article, we propose the Portiloop, a deep learning-based, portable and low-cost closed-loop stimulation system able to target specific brain oscillations. We first document open-hardware implementations that can be constructed from commercially available components. We also provide a fast, lightweight neural network and an exploration algorithm that automatically optimizes the network model to the desired brain oscillation. Finally, we validate the technology on a challenging test case of real-time sleep spindle detection, with results comparable to off-line expert performance. Software and plans are available to the community as an open science initiative to encourage further development and advance closed-loop neuroscience research")

(*Continued on next page*)

continued from previous page

ID	Reviewer's comment	Answer
2-2	Reduced ANNs for maximizing Recall and Precision are not developed or analyzed. No reduced ANN matched SpindleNet's Recall (% true positives). Reduced ANNs all had superior Precision (% positives found). Why? Three reduced models strike a balance between Recall and Precision with f1 scores of 0.61, but no model variations achieved the performance of expert raters. Why? All the reduced models perform essentially the same.	Please note that no model can achieve the performance of expert raters for several reasons, most notably that the expert ratings are what we use as our ground truth and thus achieving their performance is statistically impossible. But furthermore, they have a fairly low inter-rater agreement, which means that their ratings (that is the closest we have to ground truth labels) are in fact themselves pretty noisy. We have improved our explanation of the challenges involved in labelling spindles (see Section entitled "Offline sleep spindle detection for labeling and performance comparison").<br>We have also improved our explanation of the f1 score. ("Once trained, the success of an algorithm on classifying previously unseen data can be quantified using the f1-score, which is a widely used metric to quantify an average of recall (i.e., success in detecting events) and precision (i.e., the proportion of detected events that are correct), see Equation 1."; a Figure 9 caption also describes the trade-off with an illustration, "This provides a visualization of possible trade-offs in terms of how many spindles we want to stimulate (recall) versus how sure we want to be that all stimuli are relevant (precision). In terms of f1-score, the best such trade-off is attained at a threshold of 0.84 with our model, yielding a precision and a recall of 0.71.")<br>Recall and Precision are meaningless metrics when considered independently from each other: if we predicted that everything is a sleep spindle we would obtain a Recall of 100%, and if we predicted that only one data point for which we are 100% sure to be in a sleep spindle is indeed in a sleep spindle while everything else isn't we would obtain a Precision of 100%. The f1-score, on the other hand, is a trade-off between these two metrics, and it makes some sense to maximize it, contrary to the Recall or Precision.<br>We do explore the optimal tradeoffs between Recall and Precision in-depth in the submission, both in terms of detection and real time stimulation (Figures 9, and in Supplementary Information show different aspects of this).<br>Concerning the reduced models, we conduct an ablation study that shows the 1-input does indeed perform the same as the 2-input (which is why we select the reduced 1-input model, see Table 1 rows (3) and (6)). Then, we perform other ablations on this model only. The reduced model trained without time-dilation performs much worse (f1=0.47, see Table 1 row (7)) than the selected model (f1=0.61, Table 1 row (6)). The subsequent ablation is more subtle and shows that the "younger" group (p1) contributes more to training than the "older" group (p2) (see Table 1, rows (8-10)). We have clarified these observations in the text.

(*Continued on next page*)

continued from previous page

**ID**	**Reviewer's comment**	**Answer**
2-3	More than half the figures (Figures 1, 3-7, 9) only highlight processes and methods. The rest of the figures cannot be interpreted without the main text. Therefore, the scope of analyses and presentation of the results required significant improvement.	Also at the suggestion of another reviewer, we have reduced the number of figures and moved some to Supplementary Information. We have also adjusted the captions to be clearer as to the purpose of each figure, and we believe they will be easier to interpret when placed in the context of the text with their captions (as opposed to the review format). We have considerably restructured the manuscript to clarify the scope and aims (please see Introduction, which has been largely re-written). The figures do highlight processes and methods for the most part, as these are the main advances in this interdisciplinary work; the spindle application is intended as a case study and validation, and to demonstrate the behaviour of the system with respect to performance trade-offs.
2-4	Table 1. Please spell out IExp, as this term / abbreviation is not used in the text	Thank you we have replaced it with Inter-rater agreement
2-5	No stimulation is actually conducted in this study. Do the Authors mean actuate?	We agree that "actuate" would be a more general term. However, given the proposed application in brain stimulation and usage in that field, we prefer to keep the stimulation terminology.

References
