# Supplementary material for: The Portiloop: A deep learning-based open science tool for closed-loop brain stimulation
Source: PLoS One. 2022 Aug 22;17(8):e0270696. doi: 10.1371/journal.pone.0270696 (PMC9394839; doi:10.1371/journal.pone.0270696)
Supplement: Attachment — Submitted filename: answer_to_reviewers.pdf [file pone.0270696.s003.pdf]

ANSWERS TO THE REVIEWERS' COMMENTS

We sincerely thank the editor and the reviewers for their time and comments on our last revision, and we express our appreciation for their contribution in making this manuscript better and clearer. We hope that our latest changes are sufficient for publication.

For the editor and reviewers' convenience, we provide a detailed answer to each comment, and we coloured the modified text in the manuscript.

ID	Reviewer's comment	Answer
**Editor**		
1-1	Please, provide actual examples of the EEG and how Portiloop classifies events, in this case, sleep spindles within the time series, and how this classification compares to expert manual classification.	The examples requested were present in the Supplementary Material. We have moved the content to the main paper with what is now Figure 2.
1-2	Abstract. Please, define and mention MODA in the Abstract.	Done.
1-3	Please, consider removing Figures 3. In Figure 6, it will help to see the raw and processed signal, along with its envelope, and this could be integrated into Figure 2.	We have removed Figure 3 (which was added to answer one of the reviewer's concerns) and we have instead put an up-to-date figure of the Portiloop UI on the projects' website. We have also merged the spindle examples annotations into Figure 2 as requested, and added a legend.
1-4	Table 2. The absence of visuals to support Portiloop's performance is a concern. The only figure that shows Portiloop's processed EEG are in Figure 2. This paper needs a figure showing how Portiloop's different variations detect spindles in EEG compared to the nominal ground truth, the MODA dataset. MODA vs. 2-input Portiloop vs. 2-3 variants of Portiloop that favor speed over classification accuracy.	We thank the editor for the comment, and we agree that visual support is important. It is worth noting that spindles have a lot of variance between individuals or even in time for a single individual. Therefore we tried to supply an average example of representative detection, which we have moved from the Supplementary Material to Figure 2.
1-5	This paper needs a figure showing how Portiloop's different variations detect spindles in EEG compared to the nominal ground truth, the MODA dataset. MODA vs. 2-input Portiloop vs. 2-3 variants of Portiloop that favor speed over classification accuracy	The differences in terms of classification accuracy between these models are so tiny that any visual difference would be due to random ANN initialization/training or cherry picking (Figure 4 shows that most Pareto optimal models have roughly the same f1-score). In fact, according to Table 1 and the ablation study, there is even no significant difference between the models using 1 and 2 inputs in terms of classification accuracy: the 2-input model just takes longer to execute, which is why we selected the 1-input model. Note that in any case the corresponding difference in hardware delay (20ms) is negligible compared to the ANN software delay (about 250ms). In other words, the exploration does not affect model accuracy, but rather the amount of resources used on the board. We have clarified this point in the Supplementary Material, where we have moved information related to model optimization (see the following answers for details).

(*Continued on next page*)

continued from previous page

ID	Reviewer's comment	Answer
1-6	Table 2. Too many abbreviations and undefined terms in Row Set 2 – on-line detection. Figures and tables should be interpretable without the main text. Therefore, the table needs a legend to describe, at a high level, what is meant by 1- and 2-inputs, ablation, td, p1, so forth.	We believe the editor means Table 1. We have therefore extended the caption of Table 1 to clarify these abbreviations.
1-7	Figure 7. As is, Figure 7 is abstracted from real run / compute times. It would helpful to contrast the classification performance before and after optimization. What is the trade-off between compute time and classification with a high software and hardware cost (without PMBO), versus a low software and hardware cost (with PMBO). Show EEG output of two ANNs off and on the Pareto front.	While we do agree that our "hardware cost" is a proxy for the actual computation time and use of resources on FPGA, it was a necessary approximation since hardware synthesis on FPGA requires 2 hours of computation just to generate the bitstream, and longer to run performance tests, which is not feasible for runs over thousands of data points. The Pareto curve represents in fact the trade-off: the software cost is (1 - f1-score), meaning that the lower it is, the better the classification accuracy, while the hardware cost is the number of parameters of the ANN, each of which adds to the computation and storage requirements of the model. By looking at the Pareto front we can find all the configurations that trade off accuracy (f1-score) for computation (number of parameters). All the points that are not on the Pareto front are "dominated", meaning that there exists a configuration that is either better in terms of f1-score with the same number of parameters, better in terms of number of parameters with the same f1-score, or better in both metrics. Therefore, the Pareto front filters all solutions that are not optimal with respect to either metrics. It is worth noting that this trade-off applies to the average performance of the ANN and would be fundamentally impossible to see using two randomly selected example spindles, which is why we rely on the f1-score, which gives us a scalar aggregate value of performance. We have updated the paragraph describing Figure 4 to clarify the use and interpretation of the Pareto front.
1-8	Figure 8. Figure 8 needs to be rotated clockwise 90 degrees.	Done.
1-9	Figure 9. It's not clear what score and threshold mean in this figure without referencing Figure 2 and the text. Figures should be mostly interpretable in isolation. Please, show a panel (as in Fig. 2) with the EEG colored and annotated to show what are true positives, false positive, etc. for stimulation timestamps. It may also help to see an example of a delay relative to the spindle (and its duration).	We have appended "on the ANN output value" to "Evolution of the stimulation performance with respect to the chosen detection threshold" to make it clear what the threshold refers to. Concerning the scores, we believe most readers are familiar with f1-score, precision and recall, but we added a pointer to the definitions provided in equations (6), (7) and (8) of the Supplementary Information in Section 2.3 for readers who are less familiar with these terms. A visual examples of delays/duration/etc is now provided in the new Figure 2.

(*Continued on next page*)

continued from previous page

ID	Reviewer's comment	Answer
1-10	Please, consider whether you could bring the main text word count closer to 4000 or 5000 words and push more technical details to the Supplement or the Github repo.	We have done our best to reduce the length of the paper. We have moved several sections to the Supplementary Material, as suggested (e.g. 4.1 and 4.2). Because of the interdisciplinary nature of this work, it is challenging to further reduce the length while still being sufficiently thorough and informative to be interesting to readers from both disciplines. We note that the most recent article's word counts range from 6500 to 7500. The total word count of this article after reductions, excluding abstract, references, captions, and acknowledgements, is now 7345.
**Reviewer #1**		
The authors have done a good job with the revision. Objectives are laid out much clearer, and a number of well-made figures help the reader, and there is a comment about how to obtain such a device.		
2-1	My main concern with the manuscript, however, has not been adressed - it is still clearly too long to be read by a majority of the scientific community. While a part of the Methods section has been shifted to the Supplement, the word count for the main text is still between 9000 and 10000. I think the chances of having people actually read, and cite, the article would be much higher if the authors could bring the main text word count closer to 4000 or 5000 words and push more technical details to the Supplement or the Github repo. Also, the authors should be aware that the target audience are probably experimental neuroscientists more than computer scientists or electrical engineers, so a bit more information about the example study (sleep spindles) and a bit less technical detail would probably be appreciated.	We thank the reviewer for the comments, and we have done our best to reduce the lenght of the paper, pushing Sections 4.1 and 4.2 to the Supplementary Material, as well as other results related to optimization. We also refer the reviewer to answer 1-10, which covers the same issue.
**Reviewer #2**		
This design paper covers Portiloop, a closed-loop and lightweight tool for event classification in timeseries using deep learning. The paper uses detection of sleep spindles from a public dataset, MODA, as a first test case and proof of concept. The manuscript, while dense in text, adequately describes the motivation and methods for constructing Portiloop. However, the results are left mostly in text form. Nontechnical readers will benefit from seeing actual examples of the EEG and how Portiloop classifies events, in this case, sleep spindles within the time series, and how this classification compares to expert manual classification. Therefore, my remaining concerns for this manuscript are in its presentation of the results. Some figures show flow chart of methods adequately described with text alone, and some figures need to show real examples of classified EEG data.		
3-1	Abstract. I think it will help to define and mention MODA in the Abstract to give it a search link to a term relevant to sleep researchers.	Done
3-2	Figures 3 and 6. I agree with Reviewer 1's previous concern of too many figures. Figures 3 and 6 appear unnecessary. However, in Figure 6, it will help to see the raw and processed signal, along with its envelope, and this could be integrated into Figure 2.	We have removed Figure 3 and modified Figure 6 (see response 1-3)

(*Continued on next page*)

continued from previous page

ID	Reviewer's comment	Answer
3-3	Table 2. The absence of visuals to support Portiloop's performance is a concern. The only figure that shows Portiloop's processed EEG are in Figure 2. This paper needs a figure showing how Portiloop's different variations detect spindles in EEG compared to the nominal ground truth, the MODA dataset. MODA vs. 2-input Portiloop vs. 2-3 variants of Portiloop that favor speed over classification accuracy.	We invite the reviewer to look at responses 1-4 and 1-5. Fundamentally, it is very hard to show the differences in terms of detection, except on aggregate values like the f1-score computed over the validation part of the MODA dataset, which is one of the selection metrics for the Pareto points. We argue that the Pareto curve in Figure 6 is showing what the reviewer is requesting: the quality of the detection is shown by the software cost (which is 1 - f1-score), and the hardware use is expressed by the hardware cost. In terms of actual processing time, all Portiloop variants perform inference in 20 or 40 ms, which do not impact the overall detection time since the ANN latency is much larger, around 250 ms. So the Pareto curve shows the trade-off in terms of f1-score and hardware use on the board, rather than execution speed. The trade-off in terms of detection latency (the previously mentioned 250 ms) and accuracy (the f1-score) is given by the threshold parameter, and it is shown in Figure 8.
3-4	Table 2. Too many abbreviations and undefined terms in Row Set 2 – on-line detection. Figures and tables should be interpretable without the main text. Therefore, the table needs a legend to describe, at a high level, what is meant by 1- and 2-inputs, ablation, td, p1, so forth.	We believe the reviewer means Table 1. We have therefore extended its caption according to this comment.
3-5	Figure 7. As is, Figure 7 is abstracted from real run / compute times. I think it would helpful to contrast the classification performance before and after optimization. What is the trade-off between compute time and classification with a high software and hardware cost (without PMBO), versus a low software and hardware cost (with PMBO). Show EEG output of two ANNs off and on the Pareto front.	The classification performance is given by the f1-score, which is a metric for optimization, while the compute time is more or less constant as most operations are ran in parallel. The trade-off is more on the board resources (larger models means more memory and compute used in parallel on the FPGA) which ultimately affect the cost of the device (larger FPGAs are more expensive). Differences in f1-score are not visible on a EEG trace as they are aggregate values over hundreds of spindles, and spindles have very high variability between individuals and even on the same subject over time. To avoid confusion and to shorten the paper, we have moved the optimization to the supplementary material, and we have expanded the explanations of the trade-offs. Please also look at answer 1-7.
3-6	Figure 8. Figure 8 needs to be rotated clockwise 90 degrees. Figure 9. It's not clear what score and threshold mean in this figure without referencing Figure 2 and the text. Figures should be mostly interpretable in isolation. I recommend showing a panel (as in Fig. 2) with the EEG colored and annotated to show what are true positives, false positive, etc. for stimulation timestamps. It may also help to see an example of a delay relative to the spindle (and its duration).	We have updated the figures according to the reviewer's suggestions.
